# Alterations of brain network hubs in reflex syncope: Evidence from a graph theoretical analysis based on DTI

**DOI:** 10.1002/brb3.1006

**Published:** 2018-05-23

**Authors:** Bong Soo Park, Yoo Jin Lee, Jin‐Han Park, Il Hwan Kim, Si Hyung Park, Ho‐Joon Lee, Kang Min Park

**Affiliations:** ^1^ Department of Internal medicine Haeundae Paik Hospital Inje University College of Medicine Busan Korea; ^2^ Department of Radiology Haeundae Paik Hospital Inje University College of Medicine Busan Korea; ^3^ Department of Neurology Haeundae Paik Hospital Inje University College of Medicine Busan Korea

**Keywords:** diffusion tensor imaging, graph theory analysis, syncope

## Abstract

**Objective:**

We evaluated global topology and organization of regional hubs in the brain networks and microstructural abnormalities in the white matter of patients with reflex syncope.

**Methods:**

Twenty patients with reflex syncope and thirty healthy subjects were recruited, and they underwent diffusion tensor imaging (DTI) scans. Graph theory was applied to obtain network measures based on extracted DTI data, using DSI Studio. We then investigated differences in the network measures between the patients with reflex syncope and the healthy subjects. We also analyzed microstructural abnormalities of white matter using tract‐based spatial statistics analysis (TBSS).

**Results:**

Measures of global topology were not different between patients with reflex syncope and healthy subjects. However, in reflex syncope patients, the strength measures of the right angular, left inferior frontal, left middle orbitofrontal, left superior medial frontal, and left middle temporal gyrus were lower than in healthy subjects. The betweenness centrality measures of the left middle orbitofrontal, left fusiform, and left lingual gyrus in patients were lower than those in healthy subjects. The PageRank centrality measures of the right angular, left middle orbitofrontal, and left superior medial frontal gyrus in patients were lower than those in healthy subjects. Regarding the analysis of the white matter microstructure, there were no differences in the fractional anisotropy and mean diffusivity values between the two groups.

**Conclusions:**

We have identified a reorganization of network hubs in the brain network of patients with reflex syncope. These alterations in brain network may play a role in the pathophysiologic mechanism underlying reflex syncope.

## 1 INTRODUCTION

Syncope is a symptom that presents with an abrupt, transient, and complete loss of consciousness, associated with an inability to maintain postural tone, with rapid and spontaneous recovery; its presumed mechanism is cerebral hypo‐perfusion (Shen et al., 2017). Studies of syncope have reported prevalence rates as high as 41%, with recurrent syncope occurring in 13.5% of individuals (Shen et al., 2017). Reflex syncope, previously termed neurally‐mediated syncope, is the most common subtype of syncope and comprises a number of related conditions in which neural reflexes modify heart rate and blood pressure inappropriately, resulting in loss of consciousness (Alboni et al., 2001). Types of reflex syncope include vasovagal syncope, situational syncope, carotid sinus syncope, and some cases without apparent triggers (Shen et al., 2017). Although the pathophysiologic mechanisms of reflex syncope are not fully understood, alterations in autonomic activation have been put forth as the most likely cause (Alboni & Alboni, 2017; Malamud‐Kessler, Bruno, Chiquete, Senties‐Madrid, & Campos‐Sanchez, 2016). Intermittent and sudden disruptions in the activity of the autonomic nervous system can occur, causing a sudden drop in blood pressure, heart rate, and brain perfusion (Malamud‐Kessler et al., 2016). Some evidence suggests that brain abnormalities are associated with reflex syncope, highlighting the fact that specific brain regions modulate the cardiovascular autonomic nervous system (Beacher, Gray, Mathias, & Critchley, 2009; Kim, Suh, Seo, Koh, & Kim, 2014; Shin et al., 2015).

Graph theory is a mathematical tool that allows for the analysis and quantification of brain networks (Bullmore & Sporns, 2009; Sporns, 2014). Graph theoretical analysis can delineate the whole brain as a large‐scale network consisting of nodes and edges, which reveals that the human brain has a global topology of small‐worldness, in order to maintain high global and local efficiency (Bullmore & Sporns, 2009; Sporns, 2014). On the other hand, the graph theoretical approaches can also identify highly connected regions in a brain network, so‐called hub nodes, which play central roles in integrating diverse information sources and supporting fast communication with minimal energy cost. In general, hubs are believed to function as the basis of the brain’s integrative capacity, and possess a high degree of connectivity, short neuronal path lengths, and high centrality (Bullmore & Sporns, 2009; Sporns, 2014). However, no studies have investigated brain networks using graph theory in reflex syncope patients.

Diffusion tensor imaging (DTI) is used to map and characterize the three‐dimensional diffusion of water as a function of spatial location, which can reflect microstructural tissue status and orientation (Alexander, Lee, Lazar, & Field, 2017). This is made possible because water diffusion in tissues is highly sensitive to differences in the microstructural architecture of cellular membranes (Alexander et al., 2017). This sensitivity makes DTI a powerful method for detecting microscopic differences in tissue properties (Alexander et al., 2017). Although DTI has been applied to various diseases, no studies have investigated microstructural architecture in patients with reflex syncope.

We sought to evaluate the global topology and regional hubs reorganization of brain network in patients with reflex syncope using graph theoretical analysis based on DTI. This approach has many advantages. The reproducibility of graph theory metrics from structural connectomes based on DTI is significantly more accurate than studies of functional connectivity based on electroencephalography, magnetoencephalography, or resting state‐functional MRI, which have significant variability over time and a high level of within‐subject variability (Deco, Jirsa, & McIntosh, 2011; Gleichgerrcht, Kocher, & Bonilha, 2015; Mehrkanoon, Breakspear, & Boonstra, 2014). In additional, DTI studies can focus on the quantification of intuitive measurements of axonal fibers, revealing direct structural reorganization and connection, whereas studies based on cortical thickness or volumes can only evaluate structural associations indirectly, using statistical dependence or correlation (Deco et al., 2011; Gleichgerrcht et al., 2015; Mehrkanoon et al., 2014). In addition, we investigated microstructural abnormalities of white matter using the diffusion tensor and scalar values, fractional anisotropy (FA) and mean diffusivity (MD), in patients with reflex syncope compared to healthy subjects. Our hypothesis was that there were alterations of the brain network or white matter microstructures in patients with reflex syncope.

## 2 METHODS

### 2.1 Subjects

This study was conducted with the approval of our institution’s institutional review board. This study was conducted prospectively in a single tertiary hospital. Twenty patients diagnosed with reflex syncope were recruited from March 2017 to December 2017. Only patients who satisfied the following criteria were enrolled: 1) two or more episodes of clinically diagnosed reflex syncope, confirmed by a neurologist, based on the patient’s clinical history and head‐up tilt table test, 2) absence of abnormal findings on electrocardiography, echocardiography, or electroencephalography, and 3) normal brain MRI scan, by visual inspection. These patients were free of any structural heart disease, arrhythmias, diabetes mellitus, or neurologic disease, and none were taking medications that might affect autonomic function. We also enrolled an age‐ and sex‐matched control group of 30 healthy subjects with no significant past medical, neurological, or psychiatric history.

### 2.2 Brain MRI

All subjects underwent MRI using the following imaging protocol: sagittal‐oriented 3D T2‐weighted images (TR/TE = 2500.0/244.6 ms, field of view (FOV)  = 256 × 256 mm^2^, with a 1 mm^3^ isotropic voxel size), sagittal‐oriented 3D T1‐weighted images (TR/TE = 8.6/4.0 ms, FOV = 256 × 256 mm^2^, 1 mm^3^ isotropic voxel size), and coronal‐oriented 3D fluid‐attenuated inversion recovery images (TR/TE = 4800.0/266.8 ms, FOV = 240 × 240 mm^2^, a 1 mm^3^ isotropic voxel size). In addition, DTI scans were obtained for all subjects. DTI was performed using spin‑echo single shot echo‐planar pulse sequences with a total of 32 different diffusion directions (TR/TE = 8620/85 ms, FA = 90°, slice thickness = 2.25 mm, acquisition matrix = 120 × 120, FOV = 240 × 240 mm^2^, and *b*‑value = 1,000 s/mm^2^). All of the scans were obtained using a 3.0T MRI scanner equipped with an 8‐channel head coil (AchievaTx, Phillips Healthcare, Best, The Netherlands).

### 2.3 Image processing and statistical analysis

Graph theoretical analysis was performed using DSI Studio (http://dsi-studio.labsolver.org). Nodes were defined as anatomical regions, and edges were defined by fiber density. Graph theoretical analysis was performed as follows. First, a tractography was generated from the DTI data, which entails reading and parsing DICOM files, image reconstruction to characterize the major diffusion direction of the fiber, and fiber tracking. Afterwards, the connectivity matrix was generated, which was calculated from the count of the connecting tracts. The Automated Anatomical Labeling (AAL) template was used as the brain parcellation, and every white matter fiber was evaluated to determine its extreme points. This step included acquiring a whole brain fiber track, which placed the seeding at the whole brain level, spatial normalization, and definition of the regions of interest, and building the connectivity matrix. At last, we calculated the global graph theoretical network measures from the connectivity matrix, including the mean clustering coefficient, characteristic path length, small‐worldness, global efficiency, and local efficiency, to obtain quantitative information regarding the global network properties. In addition, we also obtained measures of strength, betweenness centrality, and PageRank centrality to investigate changes in hub organization. We investigated differences in the graph theoretical network measures between patients with reflex syncope and the healthy subjects. A *p*‐value <0.05 was considered significant for all calculations. Comparisons were analyzed with a Student’s t test. All of the statistical tests were performed using MedCalc^®^ (MedCalc Software version 17.8, Ostend, Belgium).

To perform tract‐based spatial statistics analysis (TBSS) analysis, all raw DTI data were preprocessed with FSL (http://www.fmrib.ox.au.uk/fsl). First, eddy current distortions and head motion were corrected by spatially normalizing all the diffusion‐weighted images. Afterwards, skull‐stripping was applied to exclude nonbrain tissues and regions. At last, we computed the diffusion tensor, as well as the scalar measures, including fractional FA and MD values (FA and MD values were analyzed with protocols provided by TBSS). We normalized individual FA volumes of the two groups to the MNI template space via affine registration. The aligned FA images were averaged to yield a mean FA image and then thinned to create the FA skeleton of the mean FA image. The skeleton represented the common tract pattern of all participants from the two groups. The FA threshold (0.2) was then set on the skeleton to exclude gray matter and cerebral spinal fluid from the final analysis. The FA image for each subject was then projected onto the skeleton. The significance threshold for between‐group differences was set at *p *<* *0.05 using threshold‐free cluster enhancement in the FSL “randomize” permutation‐testing tool (5000 permutations). Regional FA differences were then localized according to the probabilistic Johns Hopkins University White Matter Atlas. Group comparisons of the MD images were performed in a similar manner.

Categorical variables are presented in terms of both frequency and percentage. Numerical variables are presented as mean ± standard deviation.

## 3 RESULTS

### 3.1 Demographic and clinical characteristics of subjects

Of the 20 patients with reflex syncope, 10 (50%) were men and 10 (50%) were women. The mean age was 37.4 ± 14.9 years. Among the 30 healthy control subjects, 15 subjects (50%) were men and 15 subjects (50%) were women. The mean age was 37.4 ± 6.7 years. All healthy subjects had a normal neurological examination and a normal brain MRI on visual inspection. The age and sex of the healthy subjects were similar to those of the reflex syncope patients (*p *=* *0.9957 and *p *=* *1.000, respectively).

### 3.2 Measures presenting topology and hubs organization

The measures of global topology, such as mean clustering coefficient, characteristic path length, small‐worldness, global efficiency, and local efficiency, in patients with reflex syncope were not different from those in healthy subjects (Table 1). However, there were significant differences in the reorganization of hubs organization in the patients with reflex syncope compared to healthy subjects (Table 2). The strength of the right angular, left inferior frontal, left middle orbitofrontal, left superior medial frontal, and left middle temporal gyrus in the patients with reflex syncope were lower than those in healthy subjects. In addition, the betweenness centrality of the left middle orbitofrontal, left fusiform, and left lingual gyrus in the patients with reflex syncope were lower than those in healthy subjects. Moreover, the PageRank centrality of the right angular, left middle orbitofrontal, and left superior medial frontal gyrus in the patients with reflex syncope were lower than those in healthy subjects.

**Table 1:** Global network measures of topology in patients with reflex syncope and healthy subjects

Network measures	Patients with reflex syncope (*n *= 20)	Healthy subjects (*n *= 30)	*p*‐value
Mean clustering coefficient	0.1047 ± 0.0344	0.1057 ± 0.0444	0.9334
Characteristic path length	3.9390 ± 0.4198	4.1707 ± 0.4552	0.0754
Small‐worldness	0.0732 ± 0.0269	0.0763 ± 0.0369	0.7477
Global efficiency	0.9162 ± 0.0703	0.9431 ± 0.0887	0.2611
Local efficiency	118.1547 ± 15.8380	118.7017 ± 18.9259	0.9155

**Table 2:** Regions with altered hubs organization in patients with reflex syncope compared to healthy subjects

Region	Patients with reflex syncope (*n *= 20)	Healthy subjects (*n *= 30)	*p*‐value
Strength			
Rt. angular gyrus	2.9500 ± 2.7999	6.1000 ± 3.1500	0.0127
Lt. inferior frontal gyrus	14.1500 ± 4.4988	18.3333 ± 8.4622	0.0486
Lt. middle orbitofrontal gyrus	10.3000 ± 4.8569	14.1667 ± 7.4096	0.0454
Lt. superior medial frontal gyrus	45.9500 ± 10.3795	53.3667 ± 10.2771	0.0163
Lt. middle temporal gyrus	24.1500 ± 8.1903	30.5333 ± 9.6338	0.0188
Betweenness centrality			
Lt. left middle orbitofrontal gyrus	136.3228 ± 125.3335	240.4542 ± 173.8067	0.0440
Lt. left fusiform gyrus	165.9320 ± 133.1419	251.5448 ± 138.6157	0.0347
Lt. lingual gyrus	310.8274 ± 297.8339	525.6408 ± 337.6408	0.0252
PageRank centrality			
Rt. angular gyrus	0.0027 ± 0.0012	0.0043 ± 0.0024	0.0079
Lt. middle orbitofrontal gyrus	0.0057 ± 0.0019	0.0076 ± 0.0035	0.0384
Lt. superior medial frontal gyrus	0.0193 ± 0.0036	0.0214 ± 0.0036	0.0497

### 3.3 Analysis of DTI scalar values

**Figure 1:**
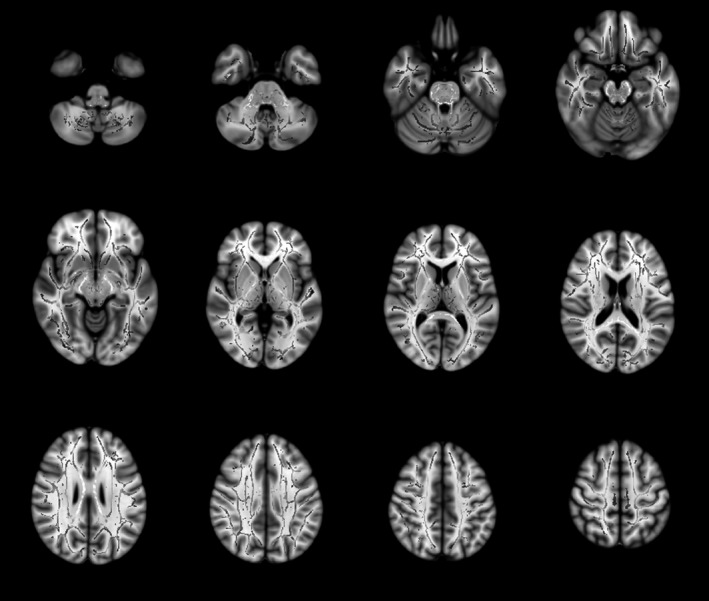
Tract‐based spatial statistics analysis of reflex syncope patients and healthy controls. Throughout the brain, no differences were detected in fractional anisotropy (FA) or mean diffusivity (MD) between patients with reflex syncope and healthy subjects

Analysis of the DTI scans showed no significant differences in FA and MD scalar values in patients with reflex syncope and healthy controls (Figure 1), indicating that brain white matter is similar in the two groups.

## 4 DISCUSSION

To our knowledge, this is the first study to explore the topological organization of brain networks in patients with reflex syncope. We calculated centrality measures for various regions in the brain and discovered that many, predominantly in the frontal lobe, were significantly decreased in patients with reflex syncope compared to healthy subjects, which suggested that there was a reorganization of the brain network hubs in patients with reflex syncope. These alterations in brain network may play a role in the pathophysiologic mechanism underlying reflex syncope.

Although the pathophysiologic mechanism of reflex syncope is not well understood, especially the afferent part of the reflex, the efferent part of the reflex seem to be elucidated with the rapid decrease in heart rate and blood pressure by change of autonomic nervous system control (Alboni & Alboni, 2017). The ventricular theory, which posits that an attempt to increase cardiac output during conditions of low venous return and ventricular filling leads to pronounced pressure transients in the myocardial wall, has been accepted (Mosqueda‐Garcia, Furlan, Tank, & Fernandez‐Violante, 2000). These pressure transients are relayed to the brain and lead to a burst of parasympathetic vagal activity, causing rapid bradycardia and peripheral vasodilatation, thereby inducing syncope (Mosqueda‐Garcia et al., 2000). Therefore, alterations of autonomic regulation of the brain could be crucial for elucidating the fundamental mechanisms of reflex syncope. In a specific manner, we found that patients with reflex syncope had reduced centrality measures in the cerebral cortex, predominantly within the frontal lobe. Thus, one interpretation of our findings is that the frontal lobe plays a critical role in autonomic regulation. Several studies have described results consistent with our findings, suggesting that autonomic regulation occurs within the frontal lobe. First, in familial dysautonomia, a recessive genetic disorder affecting the development and function of the sensory and autonomic nervous systems, voxel‐wise analysis of individuals with this disorder revealed brain atrophy within the frontal lobe (Axelrod et al., 2010). Second, postural tachycardia syndrome is a form of dysautonomia. A study using voxel‐based morphometry revealed diminished gray matter volume within the frontal gyrus in patients with postural tachycardia syndrome (Shin et al., 2015). Third, chronic complex regional pain syndrome is a debilitating pain condition accompanied by autonomic abnormalities. A previous report using functional MRI demonstrated that the chronic complex regional pain syndrome was associated with a diffuse frontal lobe hyperactivity, which was diminished or decreased with sympathetic nerve blocks (Apkarian, Thomas, Krauss, & Szeverenyi, 2001). Another study with chronic complex regional pain syndrome using voxel‐based morphometry also showed gray matter atrophy in the frontal lobe (Geha et al., 2008). Fourth, a previous study using concurrent microelectrode recordings of sympathetic outflow to either muscle or skin and functional MRI concluded that the frontal lobe is involved in the generation of sympathetic nerve activity (Macefield, James, & Henderson, 2013). At last, there are anatomical connections between the frontal lobe and the nucleus of the solitary tract/hypothalamus, which are well known regions for regulation of the autonomic nervous system (McKlveen, Myers, & Herman, 2015), These previous reports, taken together with the present study, reveal that the frontal lobe is a key brain region for autonomic control, and suggest a link between dysregulated physiologic reactions arising from compromised frontal autonomic control and increased vulnerability to reflex syncope.

However, recently Blanc & Benditt (2016) proposed a new hypothesis to try to explain and evolution of reflex syncope, “the brain self‐preservation theory”. According to this theory, reflex syncope appears to be a protective mechanism for the brain. Under certain circumstances, the cerebral blood flow can decrease; the faint causes the body to take on a gravitationally neutral position and thereby provides a better chance of restoring brain blood supply and preserving long‐term brain function. Thus, it could be possible that our results may be produced by cerebral hypo‐perfusion insults. Repeated hypo‐perfusion insults could produce reorganization of network hubs in the brain network of patients with reflex syncope. Several previous reports demonstrated various structural abnormalities of the brain in patients with reflex syncope. Beacher et al., (2009) used voxel‐based morphometry to demonstrate that patients with neuro‐cardiogenic syncope had significant gray matter volume reductions in the medulla and midbrain. Another study observed right insular atrophy in patients with neuro‐cardiogenic syncope with positive response to the head‐up tilt test, implicating the role of right insular dysfunction in the pathophysiologic mechanism underlying neuro‐cardiogenic syncope (Kim et al., 2014). In addition, we previously demonstrated that the cortical thickness of orbitofrontal, pericalcarine, postcentral, inferior temporal, and lateral occipital cortex significantly changed in patients with orthostatic hypotension but not in patients with postural tachycardia syndrome (Shin et al., 2015). These discrepancies could be caused by the different subjects and imaging methods used, or produced by the different vulnerable brain structures to hypo‐perfusion according to various subjects.

With DTI scans, FA and MD values can be obtained to assess the changes in white matter tracts. In general, FA and MD values are sensitive to microstructural changes in white matter and the microstructural architecture of cellular membranes, respectively (Alexander et al., 2017). Although the direct comparison of FA and MD values at each voxel across different groups can reveal local changes, the results of this method can be significantly and adversely affected by unavoidable registration errors and noise (Faria et al., 2010). Thus, we used the TBSS method, which can reduce these effects by projecting the volumetric data onto a white matter skeleton (Smith et al., 2006). However, in contrast to alterations of brain networks in patients with reflex syncope, we found the FA and MD values of the white matter in the patients with reflex syncope were not different from those in healthy subjects. A plausible explanation for this finding is that the changes of brain network are more sensitive than the structural changes of white matter.

There are several limitations to this study. First, we only investigated 20 patients with reflex syncope. Further studies with larger sample sizes may be needed. Second, we were unable to apply multiple test corrections in the analysis. Because we divided the whole brain into 90 subregions based on the AAL atlas to construct the brain structural network, the appropriate corrected *p*‐value for significance was = 0.00056 (0.05/90, Bonferroni correction) in the analysis. However, the statistical significance with a *p*‐value <0.00056 was too great to apply in the analysis. It could be possible that our results represent false positive due to small sample size without multiple corrections. Thus, we calculated the power of this study using the Power and Sample Size Program (http:// ps‐power‐and‐sample‐size calculation. software. informer.com/download/). It revealed that the statistical power of this study was sufficient to exclude type 1 error (all of them had more than 80%). Third, the major limitation of this study was that we could not identify the causal relationship between changes in the organization of the hubs of brain network and reflex syncope. Alterations of brain network may represent a preexisting vulnerability to reflex syncope, whereas we cannot rule out the possibility that these changes are the result of decreased cerebral perfusion or hypoxic episodes.

## 5 CONCLUSIONS

We have described here, for the first time, reorganization of the network hubs in the brain network of patients with reflex syncope. Such alterations may play a role in the pathophysiologic mechanism underlying reflex syncope.

## CONFLICT OF INTEREST

There are no conflict of interests to declare.
